# The Willingness to Pay for Vaccination against Tick-Borne Encephalitis and Implications for Public Health Policy: Evidence from Sweden

**DOI:** 10.1371/journal.pone.0143875

**Published:** 2015-12-07

**Authors:** Daniel Slunge

**Affiliations:** Department of Economics, University of Gothenburg, Gothenburg, Sweden; University of Maryland, College Park, UNITED STATES

## Abstract

The increasing incidence of tick-borne encephalitis (TBE) in Sweden and several other European countries has sparked a discussion about the need for a public vaccination strategy. However, TBE vaccination coverage is incomplete and there is little knowledge about the factors influencing vaccination behavior. Based on a survey of 1,500 randomly selected respondents in Sweden, we estimate vaccination coverage in areas with different TBE risk levels and analyze the role of vaccine price and other factors influencing the demand for vaccination. First, we find that the average rate of TBE vaccination in Sweden is 33% in TBE risk areas and 18% elsewhere. Income, age and risk-related factors such as incidence of TBE in the area of residence, frequency of visits to areas with TBE risk, and experience with tick bites are positively associated with demand for TBE vaccine. Next, using contingent valuation methodology, we estimate the willingness to pay for TBE vaccination among the unvaccinated respondents and the effect of a possible subsidy. Among the unvaccinated respondents in TBE risk areas, we estimate the mean willingness to pay for the recommended three doses of TBE vaccine to be 465 SEK (approximately 46 euros or 40% of the current market price). We project that a subsidy making TBE vaccines free of charge could increase the vaccination rate in TBE risk areas to around 78%, with a larger effect on low-income households, whose current vaccination rate is only 15% in risk areas. However, price is not the only factor affecting demand. We find significant effects on vaccination behavior associated with trust in vaccine recommendations, perceptions about tick bite-related health risks and knowledge about ticks and tick-borne diseases. Hence, increasing knowledge and trust, as well as ease of access to vaccinations, can also be important measures for public health agencies that want to increase the vaccination rate.

## Introduction

An increasing number of tick-borne encephalitis (TBE) cases have initiated a discussion about the need for a public vaccination strategy, potentially including a vaccine subsidy, in Sweden and other European countries [1–4]. This study estimates the willingness to pay (WTP) for TBE vaccination and the effect of a possible TBE vaccine subsidy on vaccination rates in Sweden. We also estimate the current vaccination coverage in areas differing in TBE incidence and analyze the role of income, risk behavior and other factors influencing vaccine demand.

TBE is caused by the TBE virus, a flavivirus transmitted to humans by ticks, which can cause severe infection of the central nervous system. Around 40% of those infected by the European subtype of the virus suffer from serious long-term or permanent sequelae [5]. Elderly people tend to get the most serious sequelae, but lately it has been recognized that young children also can get serious and long-term sequelae from TBE [6]. In Sweden and several other European countries, risk areas are expanding and incidence is increasing [7, 8]. There is no treatment, but effective vaccines are available [9].

WHO recommends that vaccination should be offered to all age groups in areas with an incidence higher than 5 annual cases per 100,000 persons [9]. Austria is the only country that has implemented a TBE vaccination program targeting the whole population. As a result, vaccination coverage in Austria increased from 6% in 1980 to 85% in 2011 and the number of TBE cases decreased from almost 700 in 1979 to less than 100 per year in the period 2000–2005 [10–12]. Several countries, including Slovenia, Latvia, and Finland, have experimented with targeted vaccination campaigns in which the price of the vaccine has been reduced for specific target groups and areas [13–15].

In Sweden, there has been a marked increase in the number of reported TBE cases during the last two decades and, subsequently, an increase in the number of TBE vaccine doses sold in Sweden, from below 100,000 doses a year in the early 1990s to 500,000–600,000 doses a year since 2006 (S1 Fig). TBE vaccination is recommended by Swedish health authorities for people spending time outdoors in TBE risk areas. However, it is not included in the national vaccination program [4].

While there are numerous studies on the willingness to pay for other vaccines [16–20], there are to our knowledge no published studies on the WTP for TBE vaccination. Hence, this study makes an important contribution to the few existing health economics studies on TBE vaccination [1–3, 21, 22]. Our analysis of the demand for TBE vaccination at current market prices complements a recent study, which estimated TBE vaccination coverage in the county of Stockholm [4]. Based on a survey of the Swedish population, we estimate vaccination coverage in areas differing in TBE incidence. Besides the variables that previously have been identified to be associated with TBE vaccination–outdoor activities in high-risk areas, age, income and country of birth [4]–we identify the role of knowledge, risk perception and trust in vaccine recommendations.

## Methodology

### 2.1. Survey instrument development and data collection

To elicit respondents’ willingness to pay for TBE vaccination, we used established contingent valuation survey methodology [23, 24]. A questionnaire was developed based on focus group discussions, two pilot tests, and key informant interviews with doctors and epidemiologists specializing in tick-borne diseases. The survey was performed under informed consent and approved by the Regional Ethical Review Board at the University of Gothenburg (decision number 544–13).

The questionnaire asked about exposure, risk perception, knowledge, and protective behavior related to ticks and tick-borne diseases, as well as socioeconomic information about the respondent and his/her household. To quantify the effect of a possible vaccine subsidy, the unvaccinated respondents were asked about their (WTP) for TBE vaccination using the following question: “*Would you vaccinate yourself or someone in your household against TBE if it cost a total of [100*, *250*, *500*, *750*, *1000] SEK for the three doses of the vaccine that protect ONE person for at least three years*?” Each respondent was presented with one of the five different prices shown in the brackets. The prices were randomly assigned to respondents so that each hypothetical price was presented to one-fifth of the unvaccinated respondents.

Because stated preference studies can be sensitive to design issues [23, 24], we used several techniques to avoid potential bias. The risk that respondents make different choices in a survey than they would in a real-life situation is usually lower for goods purchased for individual use than for goods that benefit the general public [25]. Nevertheless, we urged the respondents to answer the question as if it were a real-life situation [26, 27] and respondents were asked how certain they were about their answers [28]. We find no significant differences in results between those stating they were certain about their answer and those stating they were uncertain about their answer to the question about WTP for TBE vaccination (See Table C in S1 Text).

The survey (S2 Text) was distributed online in October 2013 to 6,000 respondents aged 18–85 years in a national internet panel representative of the Swedish population. The internet panel consists of approximately 8,000 members recruited in connection with telephone interviews with randomly sampled respondents (i.e., this is not a voluntary opt-in survey). Respondents were reminded twice to complete the questionnaire. 1,526 respondents completed the questionnaire and an additional 540 respondents answered several but not all questions, corresponding to a response rate of 25% for the whole questionnaire and 25%–34% for selected questions. Thirty-one percent of the respondents answered the questions about whether they were vaccinated against TBE.

A crucial question related to the relatively low response rate is whether those responding are more interested than the general population in TBE vaccination. Ideally, we would compare the share of TBE vaccinated respondents in our sample with the vaccination rate among the Swedish population. However, because there is no TBE vaccine register in Sweden, there are no comparative statistics on vaccination rates. A recent study of TBE vaccination rates in Stockholm County [4] finds that 53% of the population had ever received a TBE vaccine shot. Among the 415 respondents in our survey living in Stockholm County, 50% had received a TBE vaccine shot, signifying that our survey found approximately the same vaccination rate. This reduces our concerns about the response rate.

We find some statistically significant differences in socioeconomic characteristics between our survey respondents and the Swedish population. While the differences are small in magnitude, we control for their potential impact on the estimated effects of a possible vaccine subsidy by using population mean instead of sample mean values in the model used for the predictions (S4 Text).

### 2.2. Data analysis

We model the demand for a TBE vaccine as derived from the individual’s demand for health, subject to a budget constraint [29]. We propose that the demand for a TBE vaccine is a function of the price of the vaccine; the incidence of TBE in the area of residence; the behavioral risk associated with outdoor habits; experience with ticks and tick-borne diseases; knowledge about tick-borne diseases; risk perceptions related to tick bites; trust in vaccine recommendations; and socioeconomic characteristics (age, gender, income, and education). We use a binary logit regression model to study what *actually* made people get vaccinated against TBE. The dependent variable *Vaccinated* equals 1 if the respondent is vaccinated and 0 if not.

To study the *hypothetical* WTP for TBE vaccination, we also use a binary logit regression model. The dependent variable *Buy* equals 1 if the respondent states he/she would buy the vaccine at the offered price and 0 if he/she would not. Using a utility difference framework [30], we assume that respondents would buy the vaccine if it led to greater utility (welfare) relative to not buying the vaccine. With a random utility model containing a linear utility function, we calculate unvaccinated respondents’ mean WTP (*E*[*WTP*]) for a TBE vaccine as:

$$E\left[WTP\right]=\frac{\alpha +\beta \bar{z}}{\mu }$$

where α is the intercept, β is the estimated coefficient of each explanatory variable in the regression model, $\bar{z}$ is the vector of the explanatory variables, and μ is the estimated coefficient of the bid variable, or the marginal utility of income.

We use the delta method to estimate the standard error of the expected WTP. The estimated median WTP is equal to mean WTP due to the assumption of symmetric distribution in the parametric estimate. We also estimate a non-parametric mean WTP with the Turnbull estimator [31] (S1 Text).

As an objective indicator of TBE risk in different areas of residence, we use an incidence-based risk classification of Swedish postal code areas based on geographical data for the 2,687 reported TBE cases in Sweden for 1986–2012 from the Swedish Public Health Agency and population data from Statistics Sweden. We calculate TBE incidence as the average number of TBE cases per 100,000 inhabitants in each three-digit postal code area during the 27-year period. Following the classification of risk areas used by many Swedish regional health authorities when producing TBE risk maps, we define “TBE risk areas” as areas where there is positive TBE incidence and there have been two or more reported cases of TBE in a three-digit postal code area during 1986–2012. We divide this broad category into “TBE low-risk areas,” defined as TBE risk areas with an incidence lower than 5, and “TBE high-risk areas,” defined as TBE risk areas with an incidence of 5 annual TBE cases or more per 100,000 inhabitants [9].

## Results

### 3.1. Descriptive statistics

The incidence of TBE varies greatly with location. For instance, 32% of our respondents live in a low-risk area and 6.5% live in a high-risk area. Among respondents’ areas of residence, we found the highest TBE incidence to be 41 TBE cases per 100,000 inhabitants, substantially exceeding the rate at which WHO recommends vaccination.

However, living in an area with high TBE risk does not necessarily imply that a respondent has a high risk of getting TBE. The variable *Outdoor in TBE risk areas* captures behavioral risk, with 37% of respondents reporting spending time in forests or other areas where there are ticks and where they know or think there is also TBE.

Tick bites are common, with 68% of the respondents reporting that they had been bitten at least once. Tick-borne disease is common as well: 45% had either had a tick-borne disease (13%) and/or a family member or close friend who had had a tick-borne disease (41%). Eight respondents (0.5%) had had TBE and 51 respondents (3%) had a family member or close friend who had had TBE.

Perceptions about health risks and trust in vaccinations also varied, with 42% of the respondents answering that tick bites constitute a rather large or very large risk to his/her health or the health of his/her family. However, 18% had low or very low trust in vaccine recommendations from health care institutions. As we will show, distrust can offset some of the effect of a subsidy on vaccination behavior.

We also identified gaps in respondents’ knowledge about TBE. The average score on the seven knowledge questions (S2 Text) was 3.8. For example, 61% of the respondents knew there is vaccine that can prevent TBE, but only 32% knew that the disease cannot be treated with antibiotics. As with trust, knowledge can affect demand for vaccination.

Table 1 provides definitions and summary statistics of the variables included in the analysis.

**Table 1:** Variable definitions and summary statistics.

Variable	Obs	Mean	S.D.	Min	Max	Definition
***Socioeconomic***						
Female	1526	0.53	0.50	0	1	1 = female respondent
Age	1526	51.4	17.0	18	80	Years
Income	1526	44.1	23.0	5	115	Household pre-tax income/month(1,000 SEK)^a^
University	1526	0.52	0.50	0	1	1 = has studied at university
Urban	1526	0.47	0.50	0	1	1 = lives in city with>50,000 inhabitants
***TBE Vaccination***						
TBE vaccinated	1526	0.24	0.43	0	1	1 = vaccinated
***TBE risk in residence area and summerhouse area***						
TBE incidence in area of residence	1526	1.11	4.05	0	41.3	TBE incidence in respondents’ residence area
TBE risk summerhouse	1526	0.17	0.37	0	1	1 = spends time in summerhouse in area with ≥2 documented TBE cases
***Behavioral risk***						
Outdoor in TBE risk area	1526	0.37	0.48	0	1	1 = spends time outdoor in TBE risk areas^b^
Risk of tick bite at work	1526	0.10	0.29	0	1	1 = risk of getting tick bite while working
***Experience with ticks***						
Tick bite ever	1526	0.68	0.47	0	1	1 = has had at least 1 tick bite in lifetime
Tick-disease experience	1526	0.45	0.50	0	1	1 = the respondent or his/her family or friend has had tick-borne disease
***Knowledge about ticks and tick-borne diseases***						
Knowledge	1526	3.81	1.79	0	7	No. of correct answers to the 7 knowledge questions
***Risk perception***						
Health risk of tick-bites	1526	0.42	0.49	0	1	1 = tick bites perceived as very or rather large risk to respondent or his/her family’s health
Low trust in vaccine recommendations	1526	0.18	0.38	0	1	1 = rather low or very low trust in vaccine recommendations from healthcare institutions

^a^ Respondents indicated their income in intervals of 10,000 SEK. The midpoint of the scale is used in the data. E.g., if 10–20,000 SEK was indicated, then 15,000 SEK is used.

^b^ Daily, weekly or 1–2 visits per month to areas where the respondent knows or thinks there is TBE.

### 3.2. Vaccination rate

We find incomplete vaccination coverage throughout Sweden, with 24% of the respondents reporting they were vaccinated against TBE. Almost 90% of these respondents indicated they had received their last shot in the last five years, but this does not necessarily imply that they were fully protected. Hence, “vaccinated” should be interpreted here as a person who has ever received a dose of TBE vaccine.

We find a vaccination rate of about 33% in TBE risk areas and 18% elsewhere. In TBE high risk areas (i.e., areas with an incidence of 5 annual TBE cases or more per 100,000 inhabitants), the vaccination rate was 55%, compared with 30% in TBE low risk areas (i.e., areas with an incidence between 0 and 5). In areas without TBE risk, i.e., areas with zero TBE incidence or where there has been only one reported TBE case ever, there is a large difference in vaccination rates between respondents living north (5%) and south (22%) of the biogeographical boundary Limes Norrlandicus. Although ticks have spread further north in Sweden in recent decades, the prevalence of ticks and tick-borne diseases north of this boundary is considerably lower than in southern Sweden [32].

### 3.3. Who gets vaccinated against TBE?

Using a binary logit regression model, we identify variables that have a statistically significant association with the probability of TBE vaccination (Table A in S3 Text). Similarly, to a previous study [4], we find that income, age and frequency of visits to forests or other areas with TBE risk are positively associated with higher vaccination probability. Having a low household income (less than 20,000 SEK pre-tax/month) is associated with a 7 percentage point lower vaccination probability compared to households with higher incomes. Being older than 65 years is associated with a 7 percentage point higher vaccination probability compared to individuals aged 31–65 years. Frequent visits to forests or other areas with TBE risk are associated with a 20 percentage point higher vaccination probability. We find no gender differences in vaccination probability. In contrast to a previous study [4], we do not find a statistically significant association between being born outside Europe and vaccination probability.

We also find that knowledge about ticks and tick-borne diseases, risk perceptions related to tick bites, and trust in vaccine recommendations are associated with the probability of being vaccinated. The vaccination probability is around 6 percentage points higher for individuals perceiving that tick bites constitute a very serious or rather serious risk to their own or their family members’ health. Very low or rather low trust in vaccine recommendations in general, not specifically linked to TBE, is associated with a 6 percentage point decrease in the vaccination probability.

In addition, vaccination behavior is positively correlated with the TBE incidence level in the respondents’ area of residence. Living in an area with a one-unit higher incidence is associated with a 1.3 percentage point higher vaccination probability. Having access to a summerhouse in a TBE risk area is associated with an 11 percentage point higher vaccination probability.

### 3.4. Willingness to pay for TBE vaccination

**Fig 1:**
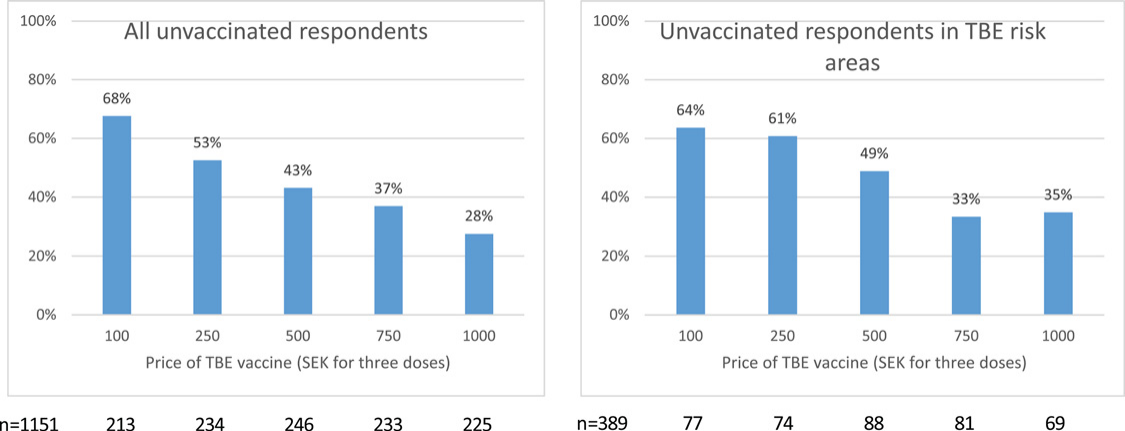
Share of unvaccinated respondents stating they would get vaccinated against TBE at different prices (SEK).

Fig 1 displays the share of unvaccinated respondents stating they would get vaccinated if the total price of the recommended three doses of vaccine was the bid price presented to them in the survey. As expected, an increasing share of the respondents state they would buy the TBE vaccine when the price of the vaccine decreases.

A rather large share (35%) of the unvaccinated respondents in TBE risk areas say they would get vaccinated at the price of 1,000 SEK, i.e., only 50 SEK (approximately 5 euros) less than the current market price. There is also a substantial share (36%) of the respondents in TBE risk areas stating they would not get vaccinated even if the price was only 100 SEK for the three doses of vaccine. In fact, 13% of the unvaccinated respondents in areas with TBE risk state that they would not get vaccinated even if the vaccine was free of charge.

This reflects that many factors besides vaccine price influence vaccination behavior. Inertia can be such a factor. When unvaccinated respondents in risk areas were asked why they were not vaccinated, 25% said they intended to get vaccinated but had not yet gotten to it and 6% responded that it was complicated and took too much time to get vaccinated. Thus, reducing the time and search costs associated with finding a vaccination provider may be more important for increasing the vaccination rate in this group than lowering the vaccine price.

Other reasons for not being vaccinated among respondents living in risk areas included: rarely visiting areas with ticks or TBE risk (24–25%), low perceived risk (15%), afraid of vaccine side effects (18%) and the vaccine costing too much (15%). As many as 26% of the respondents in risk areas state that they have never thought about getting vaccinated and 11% were not aware that a vaccine existed. This indicates that increasing the knowledge and trust about TBE risk and vaccination can be important measures for increasing the vaccination rate.

Next, we estimate mean willingness to pay among the unvaccinated respondents. Using a binary logit regression model (see Section 2.2), we find that the mean WTP for three doses of TBE vaccine is 464 SEK (95% CI 331–597 SEK) among respondents living in TBE risk areas. Among all unvaccinated respondents, mean WTP is 402 SEK (95% CI 331–474 SEK).

The parameter values for the WTP estimates are derived from the logit regression with *BUY* (respondents stating they would get vaccinated at the offered bid price) as the dependent variable (Table 2).

**Table 2:** Determinants of willingness to pay for TBE vaccination; Marginal probabilities after logit evaluated at sample means^a^.

	Not vaccinated respondents	Not vaccinated respondents	Not vaccinated respondents	Not vaccinated respondents in TBE risk- areas
	(1)	(2)	(3)	(4)
VARIABLES	Buy	Buy	Buy	Buy
Price	-0.042***	-0.043***	-0.046***	-0.042***
	(0.005)	(0.005)	(0.005)	(0.009)
Female		0.089***	0.059*	0.119**
		(0.031)	(0.034)	(0.059)
Age		0.003***	0.002**	0.002
		(0.001)	(0.001)	(0.002)
Income		0.003***	0.003***	0.002*
		(0.001)	(0.001)	(0.001)
University		-0.006	-0.026	0.030
		(0.032)	(0.033)	(0.058)
Urban		-0.052	-0.047	0.017
		(0.031)	(0.034)	(0.062)
TBE incidence in area of residence			0.001	0.002
			(0.009)	(0.011)
TBE risk summerhouse			0.039	0.026
			(0.053)	(0.076)
Outdoor in TBE risk area			0.107***	0.183***
			(0.037)	(0.060)
Risk of tick bite at work			0.101*	-0.044
			(0.059)	(0.091)
Knowledge			0.025**	-0.003
			(0.010)	(0.017)
Tick bite ever			-0.055	0.012
			(0.036)	(0.065)
Tick-disease experience			0.034	0.134**
			(0.036)	(0.060)
Health risk tick bite			0.159***	0.149**
			(0.035)	(0.058)
Low trust in vaccine recommendations			-0.161***	-0.137**
			(0.037)	(0.065)
Constant	0.688***	-0.441	-0.763**	-1.031*
	(0.116)	(0.289)	(0.329)	(0.596)
Observations	1,151	1,151	1,151	389
Pseudo R2	0,05	0,07	0,12	0,12

Robust standard errors in parentheses

*** p<0.01

** p<0.05

* p<0.1

^a^ Table B in S1 Text contains descriptive statistics of the variables included in the model.

Columns 1–3 show results for all unvaccinated respondents and Column 4 shows results for unvaccinated respondents living in TBE risk areas. The marginal probabilities represent the marginal change in the probability of buying TBE vaccination due to a marginal change in the explanatory variable, or in the case of binary explanatory variables, a change from 0 to 1.

Here, we report on variables with a statistically significant association with vaccination probabilities among unvaccinated respondents living in TBE risk areas. As expected, WTP for TBE vaccination is negatively associated with the price of the vaccine and positively associated with income. A 100 SEK price reduction increases the vaccination probability by 4 percentage points and 1000 SEK higher income increases the vaccination probability by 0.2 percentage points. We also find that the vaccination probability among women is 12 percentage points higher than among men.

Similar to the findings about who gets vaccinated at current market prices, we find a higher vaccination probability for those with frequent visits to forests or other areas with TBE risk (18 percentage points), among respondents with experience of tick-borne diseases (13 percentage points), and among those who believe that tick bites constitute a very serious or rather serious risk to their own or their family’s health (15 percentage points). The vaccination probability is 14 percentage points lower among respondents with low trust in vaccine recommendations compared to respondents with higher trust.

We also include several variables which we find are not significantly associated with vaccination probability. These are age, level of education, knowledge about tick-borne diseases, TBE incidence in the area of residence, having access to a summerhouse in a TBE risk area and living in a larger urban area (S1 Text).

### 3.5. The effects of a possible TBE vaccine subsidy

Using the regression model on WTP for TBE vaccination, we predict the demand for TBE vaccination at different prices among the unvaccinated respondents living in TBE risk areas (S4 Text; S2 Fig). The price considered is for the three doses of TBE vaccine recommended for disease protection.

Demand increases with reduced prices; the average marginal effect per SEK of subsidy is 0.065 percentage points. We predict that, with a full subsidy (i.e., making the TBE vaccination free of charge), 68% (CI95 59–77%) of the currently unvaccinated respondents in TBE risk areas would get vaccinated. With the estimated current vaccination rate of 33% in TBE risk areas, such a subsidy could increase the vaccination rate by an additional 45 percentage points to 78%. Similarly, a 50% subsidy reducing the price of three vaccine doses to 525 SEK is predicted to increase the vaccination rate among unvaccinated respondents in TBE risk areas to 46% (CI95 41–52%), resulting in a total vaccination rate of approximately 64% in TBE risk areas.

**Fig 2:**
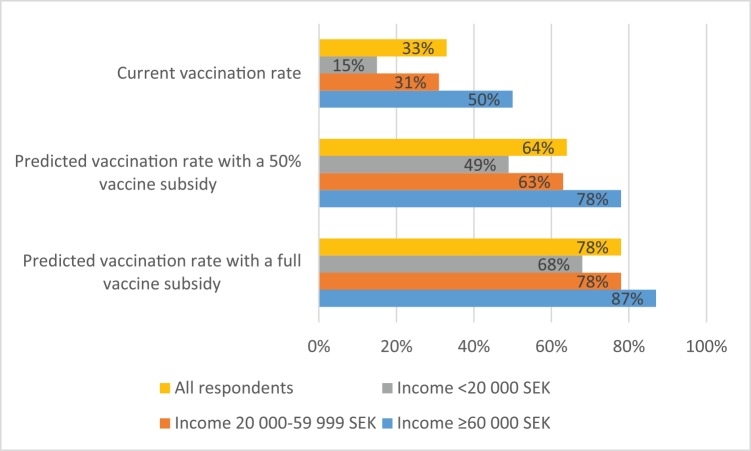
Predicted vaccination rates with a TBE vaccine subsidy in TBE risk areas (%). All respondents and respondents in different income groups (pre-tax monthly household income in SEK).

We also find that a subsidy would have a relatively larger effect on the vaccination rate among low-income households than among mid- and high-income households. In TBE risk areas, there is a large difference in vaccination rates across households with different income levels. While high-income households (with a monthly pre-tax income above 60,000 SEK) have a vaccination rate of 50%, mid-income households (20,000–60,000 SEK) have a vaccination rate of 31%, and low-income households (earning less than 20,000 SEK per month) have a vaccination rate of only 15%. We predict that a full subsidy would increase the vaccination rates in TBE risk areas to approximately 68% among low-income households, 78% among mid-income households, and 87% among high-income households. A 50% subsidy would also have a relatively larger effect on the vaccination rates among low-income households than among households with higher incomes (Fig 2).

## Discussion

Our results have several implications. First, the current TBE vaccination strategy has resulted in a vaccination rate of about 33% in TBE risk areas and 18% elsewhere. This rate is considerably higher than in TBE endemic countries such as the Czech Republic (16%) and Slovenia (12%) but lower than in Austria (85%), which is the only country that has implemented a TBE vaccination program targeting the whole population, thus substantially reducing the incidence of the disease [11]. The possibility that those responding to our survey may be more concerned about ticks and TBE than survey non-responders and the general population could imply that the actual vaccination rate in Sweden is lower than our estimates. Hence, our results suggest that the rate of vaccination, especially in areas with high TBE risk, needs to increase in order to substantially reduce the incidence of TBE in Sweden.

Second, the demand for vaccination is only partly explained by risk-related factors such as incidence of TBE in the respondent’s area of residence, experience with tick bites, and frequency of visits to forests or other areas with TBE risk. Trust in vaccination recommendations, perceptions about the health risks associated with tick bites, knowledge, and ease of access to vaccination services also matter. Hence, increasing knowledge, trust and access can be important measures for public health agencies.

Third, in line with findings from studies on adoption of other types of vaccines, we find that income matters. The current market price of the TBE vaccine deters a substantial share of at-risk people with low incomes from getting vaccinated. Respondents with household pre-tax incomes below 20,000 SEK/month in TBE risk areas have a vaccination rate of only 15% and are 18 percentage points less likely to get vaccinated than those with higher incomes.

Fourth, our results indicate that a subsidy that reduces the price of TBE vaccines could substantially increase the demand. Unvaccinated respondents in TBE risk areas have a mean willingness to pay for TBE vaccination of 465 SEK (approximately 40% of the current market price). This indicates that even a partial subsidy could have a substantial effect on vaccination rates. We estimate that introducing a 50% subsidy (i.e., reducing the price from 1,050 SEK to 525 SEK) would cause almost 50% of the unvaccinated population in TBE risk areas to get vaccinated; this would increase the vaccination rate from around 33% to 64% in TBE risk areas. A full vaccine subsidy (i.e., providing vaccines for free) could increase the vaccination rate by an additional 14 percentage points. However, given that 13% of the unvaccinated respondents in TBE risk areas state they would not get vaccinated even if the vaccine were free of charge, while many respondents state other reasons for not getting vaccinated, we conclude that there is a diminishing marginal effect of a price subsidy. In order to increase the vaccination rate to above 70%, including TBE vaccination in the general vaccination program would most likely be necessary. Besides making TBE vaccination free of charge, such a measure would send a clear signal to the population living in TBE risk areas about how public health agencies value TBE risk.

## Supporting Information

S1 Fig Reported TBE cases 1956–2014 and number of TBE vaccine doses sold 1992–2012 in Sweden. (EPS)

S2 Fig Predicted demand for TBE vaccination at different prices among unvaccinated respondents in TBE risk areas in Sweden. (EPS)

S1 Text Willingness To Pay (WTP) estimation. (DOCX)

S2 Text Survey. (PDF)

S3 Text TBE vaccination probability estimation. (DOCX)

S4 Text Prediction of the effects of a TBE vaccine subsidy. (DOCX)
