# Supplementary material for: The Willingness to Pay for Vaccination against Tick-Borne Encephalitis and Implications for Public Health Policy: Evidence from Sweden
Source: PLoS One. 2015 Dec 7;10(12):e0143875. doi: 10.1371/journal.pone.0143875 (PMC4671589; doi:10.1371/journal.pone.0143875)
Supplement: S1 Text — (DOCX) [file pone.0143875.s003.docx]

# **S1 Text Willingness To Pay (WTP) estimation**

## Estimation of non-parametric WTP

We estimate a non-parametric mean WTP ($E[WTP_{n}$]) with the Turnbull estimator as:

$E(WTP_{n})=\sum_{j=1}^{J} \frac{n_{j}}{N_{j}}[B_{j}-B_{j-1}]$,

where J is the number of bids, B is the bid level, N_j_ is the number of respondents in the sample responding to bid B_j_ , and n_j_ is the number of respondents answering yes to bid B_j_ among N_j_ respondents. The non-parametric median WTP is the value of the bid that makes the respondents indifferent between accepting and rejecting the bid, i.e., the bid with a 0.5 probability of being accepted.

Table A displays parametric and non-parametric estimates of mean and median WTP for the three doses of TBE vaccine recommended for disease protection. The first estimate is for the whole sample of unvaccinated respondents (n=1,151) and the second for the unvaccinated respondents in TBE risk areas (n=389).

**Table A. Estimated mean and median willingness to pay for TBE vaccination in SEK among unvaccinated respondents. 95% confidence intervals in parentheses.**

|  | **All Unvaccinated Respondents** (n=1,151) | | **Unvaccinated respondents in TBE risk areas** (n=389) | |
| --- | --- | --- | --- | --- |
|  | *Mean* | *Median* | *Mean* | *Median* |
| **Parametric estimate** | 401 SEK  (330- 473) | 401 SEK | 465 SEK  (334- 595) | 465 SEK |
| **Non-parametric estimate** | 416 SEK | 250 SEK | 451 SEK | 250 SEK |

The median WTP non-parametric estimates are considerably lower than the estimated mean WTP. This is due to the relatively large share of respondents accepting the highest bid of 1,000 SEK for the vaccine.

## Descriptive statistics for WTP estimates

Table B includes descriptive statistics for variables in the model presented in *Table 2 Determinants of Willingness to Pay for TBE vaccination* in the main article.

**Table B Descriptive statistics for WTP estimates**

|  | **All Unvaccinated Respondents** | | **Unvaccinated Respondents in TBE risk areas** | |  |  |  |
| --- | --- | --- | --- | --- | --- | --- | --- |
|  | **1151 obs.** | | **389 obs.** | |  |  |  |
| **Variable** | **Mean** | **S.D** | **Mean** | **S.D.** | **Min** | **Max** | **Definition** |
| ***Dependent variable*** |  |  |  |  |  |  |  |
| Buy | 0.45 | 0.50 | 0.48 | 0.50 | 0 | 1 | 1=states he/she would buy vaccine at stated price |
| ***Socioeconomic*** |  |  |  |  |  |  |  |
| Female | 0.54 | 0.50 | 0.55 | 0.50 | 0 | 1 | 1=female respondent |
| Age | 50.44 | 16.81 | 49.62 | 16.55 | 18 | 80 | Years |
| Income | 42.03 | 22.04 | 41.81 | 20.98 | 5 | 115 | Monthly household pre-tax income (1000 SEK) |
| University | 0.50 | 0.50 | 0.44 | 0.50 | 0 | 1 | 1=has university degree |
| Urban | 0.45 | 0.50 | 0.37 | 0.48 | 0 | 1 | 1=lives in city with>50 000 inhabitants |
| ***TBE risk in area of residence and summerhouse area*** | | | | |  |  |  |
| TBE-incidence in area of residence | 0.65 | 2.47 | 1.90 | 3.97 | 0 | 41.30 | TBE-incidence in respondents living area |
| TBE risk summerhouse | 0.12 | 0.32 | 0.16 | 0.37 | 0 | 1 | 1=spends time in summerhouse in area with >1 documented TBE cases |
| ***Behavioral risk*** |  |  |  |  |  |  |  |
| Outdoor in TBE risk area | 0.27 | 0.45 | 0.38 | 0.48 | 0 | 1 | 1=spends time in forests in areas with TBE risk |
| Risk of tick bite at work | 0.08 | 0.27 | 0.10 | 0.30 | 0 | 1 | 1=risk to get tickbite while working |
| ***Knowledge and experience with ticks*** | | | |  |  |  |  |
| Knowledge | 3.52 | 1.78 | 3.67 | 1.74 | 0 | 7 | Nr of correct answers on the 7 knowledge questions |
| Tick bite ever | 0.63 | 0.48 | 0.70 | 0.46 | 0 | 1 | 1=has had at least 1 tick bite |
| Tick-disease experience | 0.40 | 0.49 | 0.46 | 0.50 | 0 | 1 | 1=the respondent or his/her family or friend has had tick-borne disease |
| ***Risk perception*** |  |  |  |  |  |  |  |
| Health risk tick bite | 0.36 | 0.48 | 0.43 | 0.50 | 0 | 1 | 1=tick bite perceived as very large/rather large risk to respondent or his/her family |
| Low trust in vaccine recommendations | 0.20 | 0.40 | 0.22 | 0.41 | 0 | 1 | 1=low/very low trust in vaccine recommendations from health care institutions |

## Control for uncertainty of answers

To control for a potential hypothetical bias, respondents were asked how certain they were about their answers to the WTP question. Here, in Table C, we add a dummy variable (“Certainanswers”) to the model presented in Table 2 in the main article; we find no significant differences in results for those stating they are rather certain or very certain about their answer to the question about WTP for TBE vaccination, compared to those stating they are rather uncertain or very uncertain about their answer.

**Table C Control for uncertainty of answers**

|  | (1) | (2) | (3) | (4) |
| --- | --- | --- | --- | --- |
|  | Not vaccinated respondents | Not vaccinated respondents | Not vaccinated respondents in TBE-risk areas | Not vaccinated respondents in TBE-risk areas |
| VARIABLES | *BUY* | *BUY* | *BUY* | *BUY* |
| Price | -0.0462*** | -0.0461*** | -0.0420*** | -0.0432*** |
|  | (0.00517) | (0.00519) | (0.00900) | (0.00909) |
| Female | 0.0564* | 0.0562* | 0.119** | 0.122** |
|  | (0.0336) | (0.0336) | (0.0599) | (0.0601) |
| Age | 0.00241** | 0.00241** | 0.00212 | 0.00203 |
|  | (0.00100) | (0.00100) | (0.00182) | (0.00183) |
| Income | 0.00299*** | 0.00299*** | 0.00217 | 0.00226 |
|  | (0.000763) | (0.000763) | (0.00140) | (0.00141) |
| University | -0.0263 | -0.0263 | 0.0381 | 0.0424 |
|  | (0.0336) | (0.0336) | (0.0597) | (0.0600) |
| Urban | -0.0489 | -0.0490 | 0.0173 | 0.0185 |
|  | (0.0336) | (0.0336) | (0.0632) | (0.0634) |
| TBE-incidence in area of residence | -0.000222 | -0.000232 | 0.000427 | 0.000828 |
|  | (0.00689) | (0.00689) | (0.00792) | (0.00799) |
| TBE risk summerhouse | 0.0378 | 0.0375 | 0.0268 | 0.0302 |
|  | (0.0516) | (0.0516) | (0.0789) | (0.0791) |
| Outdoor in TBE risk area | 0.106*** | 0.106*** | 0.176*** | 0.168*** |
|  | (0.0366) | (0.0367) | (0.0580) | (0.0585) |
| Risk of tick bite at work | 0.112* | 0.112* | -0.0529 | -0.0669 |
|  | (0.0606) | (0.0606) | (0.0946) | (0.0943) |
| Knowledge | 0.0270*** | 0.0270*** | -0.00352 | -0.00356 |
|  | (0.00994) | (0.00994) | (0.0178) | (0.0178) |
| Tick bite ever | -0.0597 | -0.0596 | 0.00739 | 0.00244 |
|  | (0.0367) | (0.0367) | (0.0667) | (0.0669) |
| Tick-disease experience | 0.0314 | 0.0317 | 0.139** | 0.138** |
|  | (0.0367) | (0.0367) | (0.0606) | (0.0607) |
| Health risk tick bite | 0.156*** | 0.156*** | 0.143** | 0.157*** |
|  | (0.0354) | (0.0354) | (0.0596) | (0.0604) |
| Low trust in vaccine recommendations | -0.157*** | -0.157*** | -0.127* | -0.125* |
|  | (0.0381) | (0.0381) | (0.0668) | (0.0673) |
| Certainanswers |  | 0.0114 |  | -0.122 |
|  |  | (0.0473) |  | (0.0779) |
| Observations | 1,132 | 1,132 | 381 | 381 |
| PseudoR2 | 0,12 | 0,12 | 0,12 | 0,12 |
| Standard errors in parentheses; *** p<0.01, ** p<0.05, * p<0.1 | | |  |  |

## Control for ordering effects

Besides the WTP for TBE vaccination question, the survey also included questions about WTP for a hypothetical vaccine against Lyme borreliosis as well as a choice experiment where respondents were asked to choose between recreational areas with different risks of being exposed to ticks and tick-borne diseases. In order to control for potential ordering effects, the three different stated preference questions were introduced to respondents in different choice orders. Each choice order was presented to one-quarter of the respondents (Table D).

**Table D. Order of stated preference questions as presented to the respondents**

| **Choice orders**  Order 1: CE. WTP Borrelia. WTP TBE  Order 2: WTP Borrelia. WTP TBE. CE  Order 3: CE. WTP TBE. WTP Borrelia  Order4: WTP TBE. WTP Borrelia. CE | **Key**  CE= Choice experiment  WTP Borrelia-soliciting WTP for hypothetical vaccine against lyme borreliosis  WTP TBE - soliciting WTP for vaccine against TBE |
| --- | --- |

When introducing a dummy variable for each choice order, we find a small ordering effect, significant at the 10% level among all respondents, but not among respondents in TBE risk areas (Table E). We conclude that the order in which the respondents answered the different choice questions does not significantly affect our estimated results.

**Table E. Control for ordering effects in WTP TBE vaccination estimates**

|  | (1) | (2) | (3) | (4) |
| --- | --- | --- | --- | --- |
|  | Not vaccinated respondents | Not vaccinated respondents | Not vaccinated respondents in TBE-risk areas | Not vaccinated respondents in TBE-risk areas |
| VARIABLES | *BUY* | *BUY* | *BUY* | *BUY* |
| Price | -0.0459*** | -0.0470*** | -0.0418*** | -0.0418*** |
|  | (0.00514) | (0.00519) | (0.00897) | (0.00903) |
| Female | 0.0593* | 0.0582* | 0.119** | 0.117** |
|  | (0.0333) | (0.0334) | (0.0591) | (0.0593) |
| Age | 0.00216** | 0.00214** | 0.00195 | 0.00200 |
|  | (0.000996) | (0.00100) | (0.00181) | (0.00182) |
| Income | 0.00312*** | 0.00313*** | 0.00244* | 0.00239* |
|  | (0.000760) | (0.000763) | (0.00140) | (0.00142) |
| University | -0.0256 | -0.0222 | 0.0299 | 0.0232 |
|  | (0.0333) | (0.0335) | (0.0592) | (0.0597) |
| Urban | -0.0468 | -0.0495 | 0.0174 | 0.0214 |
|  | (0.0333) | (0.0335) | (0.0626) | (0.0629) |
| TBE incidence in area of residence | 0.00140 | 0.00168 | 0.00234 | 0.00220 |
|  | (0.00683) | (0.00690) | (0.00791) | (0.00793) |
| TBE risk summerhouse | 0.0389 | 0.0428 | 0.0259 | 0.0239 |
|  | (0.0513) | (0.0516) | (0.0781) | (0.0784) |
| Outdoor in TBE risk area | 0.107*** | 0.110*** | 0.183*** | 0.179*** |
|  | (0.0363) | (0.0365) | (0.0573) | (0.0578) |
| Risk of tick bite at work | 0.101* | 0.105* | -0.0443 | -0.0444 |
|  | (0.0598) | (0.0599) | (0.0925) | (0.0927) |
| Knowledge | 0.0252** | 0.0248** | -0.00258 | -0.00304 |
|  | (0.00985) | (0.00988) | (0.0176) | (0.0176) |
| Tick bite ever | -0.0548 | -0.0557 | 0.0122 | 0.0102 |
|  | (0.0364) | (0.0365) | (0.0665) | (0.0667) |
| Tick-disease experience | 0.0345 | 0.0340 | 0.134** | 0.136** |
|  | (0.0363) | (0.0365) | (0.0601) | (0.0602) |
| Health risk tick bite | 0.159*** | 0.156*** | 0.149** | 0.149** |
|  | (0.0350) | (0.0352) | (0.0589) | (0.0593) |
| Low trust in vaccine recommendations | -0.161*** | -0.159*** | -0.137** | -0.138** |
|  | (0.0376) | (0.0378) | (0.0658) | (0.0658) |
| order2 |  | 0.0673 |  | -0.0831 |
|  |  | (0.0449) |  | (0.0804) |
| order3 |  | 0.0340 |  | -0.0150 |
|  |  | (0.0443) |  | (0.0741) |
| order4 |  | 0.0853* |  | -0.0275 |
|  |  | (0.0452) |  | (0.0767) |
| Observations | 1.151 | 1.151 | 389 | 389 |
| Standard errors in parentheses; *** p<0.01. ** p<0.05. * p<0.1 | | | | |
