# Supplementary material for: The Willingness to Pay for Vaccination against Tick-Borne Encephalitis and Implications for Public Health Policy: Evidence from Sweden
Source: PLoS One. 2015 Dec 7;10(12):e0143875. doi: 10.1371/journal.pone.0143875 (PMC4671589; doi:10.1371/journal.pone.0143875)
Supplement: S3 Text — (DOCX) [file pone.0143875.s005.docx]

**S3 Text TBE vaccination probability estimation**

In this document, we provide background information for Section *3.3.* (*Who gets vaccinated against TBE?*) in the main article. First, we display tables with the regression models and summary statistics for the variables included. Next, we show that the results do not change when age and income are coded as continuous variables. Finally, we control for possible omitted variable bias.

**TBE vaccination probability**

Table A shows the results from the logit regression with *Vaccinated* as the dependent variable. The first and second columns include the results for respondents in the whole sample (n=1,526). The first column includes only the explanatory variables that are arguably exogenous to the decision to get vaccinated against TBE. The third and fourth columns include the results for respondents living in areas with low TBE risk (n=485) and high TBE risk (n=100), respectively. The fifth column shows the results for respondents who live outside areas with TBE risk (n=941). All explanatory variables in the model are binary, except for the continuous variables *TBE incidence in area of residence* and *Knowledge*. The marginal probabilities displayed show the marginal change in the probability of choosing to be vaccinated due to a marginal change in the explanatory variables at the sample mean. For the binary explanatory variables, this represents a change from 0 to 1. For example, the probability that a person in a low-income household in a TBE low-risk area (column 3) is vaccinated against TBE is 11.5 percentage points lower than for somebody in a household with higher income in the same area. The standard error of the presented marginal probabilities is in parentheses. Summary statistics is presented in Table B.

**Table A. TBE vaccination – marginal probabilities after logit evaluated at sample means**

|  | All respondents | All respondents | Respondents living in TBE-low risk areas | Respondents living in TBE-high risk areas | Respondents NOT living in TBE-risk areas |
| --- | --- | --- | --- | --- | --- |
|  | (1) | (2) | (3) | (4) | (5) |
| VARIABLES | Vaccinated | Vaccinated | Vaccinated | Vaccinated | Vaccinated |
|  |  |  |  |  |  |
| Female | 0.009 | -0.014 | -0.038 | 0.086 | -0.004 |
|  | (0.022) | (0.022) | (0.043) | (0.153) | (0.021) |
| Age1830 | 0.001 | 0.036 | 0.176** | 0.273 | -0.020 |
|  | (0.034) | (0.036) | (0.080) | (0.200) | (0.028) |
| Age>65 | 0.104*** | 0.072*** | 0.177*** | 0.218 | 0.021 |
|  | (0.027) | (0.026) | (0.055) | (0.150) | (0.025) |
| Low income | -0.104*** | -0.070*** | -0.115* | -0.472*** | -0.023 |
|  | (0.027) | (0.026) | (0.059) | (0.117) | (0.026) |
| University | 0.061*** | 0.021 | 0.033 | 0.030 | 0.010 |
|  | (0.022) | (0.021) | (0.044) | (0.140) | (0.021) |
| Urban | 0.070*** | 0.074*** | 0.020 | 0.148 | 0.098*** |
|  | (0.023) | (0.022) | (0.044) | (0.210) | (0.021) |
| TBE incidence in area or residence |  | 0.013*** | 0.032 | 0.006 | -0.056 |
|  |  | (0.004) | (0.024) | (0.010) | (0.455) |
| TBE risk summerhouse |  | 0.112*** | 0.140** | -0.015 | 0.099*** |
|  |  | (0.031) | (0.055) | (0.171) | (0.036) |
| Outdoor in TBE risk area |  | 0.195*** | 0.197*** | 0.570*** | 0.122*** |
|  |  | (0.024) | (0.043) | (0.106) | (0.028) |
| Risk of tick bite at work |  | 0.098** | 0.120 | 0.167 | 0.075 |
|  |  | (0.043) | (0.076) | (0.175) | (0.049) |
| Knowledge |  | 0.048*** | 0.064*** | 0.052 | 0.036*** |
|  |  | (0.007) | (0.014) | (0.051) | (0.007) |
| Tick bite ever |  | 0.071*** | 0.037 | 0.255 | 0.060*** |
|  |  | (0.024) | (0.052) | (0.191) | (0.023) |
| Tick-disease experience |  | 0.030 | 0.079* | -0.004 | 0.008 |
|  |  | (0.023) | (0.045) | (0.173) | (0.023) |
| Health risk tick bite |  | 0.063*** | 0.023 | 0.003 | 0.078*** |
|  |  | (0.023) | (0.043) | (0.158) | (0.025) |
| Low trust in vaccine recommendations |  | -0.058** | -0.118** | 0.080 | -0.032 |
|  |  | (0.025) | (0.047) | (0.197) | (0.025) |
| Constant | -1.649*** | -4.410*** | -4.193*** | -4.268*** | -4.832*** |
|  | (0.141) | (0.289) | (0.504) | (1.277) | (0.407) |
| Observations | 1,526 | 1,526 | 485 | 100 | 941 |
| Pseudo R2 | 0,03 | 0,24 | 0,21 | 0,36 | 0,23 |

Standard errors in parentheses; *** p<0.01, ** p<0.05, * p<0.1

## Table B Extended summary statistics

|  | All respondents (1526 obs.) | | Respondents in low risk areas (485 obs.) | | Respondents in high risk areas (100 obs.) | | Respondents NOT in risk areas (941 obs) | |  |  |
| --- | --- | --- | --- | --- | --- | --- | --- | --- | --- | --- |
|  | Mean | S.D. | Mean | S.D. | Mean | S.D. | Mean | S.D. | Min | Max |
| TBE vaccinated | 0.24 | 0.43 | 0.29 | 0.45 | 0.55 | 0.50 | 0.18 | 0.39 | 0 | 1 |
| ***Socioeconomic*** |  |  |  |  |  |  |  |  |  |  |
| Female | 0.53 | 0.50 | 0.52 | 0.50 | 0.58 | 0.50 | 0.53 | 0.50 | 0 | 1 |
| Age | 51.36 | 16.95 | 51.00 | 17.11 | 55.05 | 15.46 | 51.15 | 16.99 | 18 | 80 |
| Age1830 | 0.14 | 0.35 | 0.14 | 0.34 | 0.06 | 0.24 | 0.15 | 0.36 | 0 | 1 |
| Age>65 | 0.30 | 0.46 | 0.29 | 0.45 | 0.40 | 0.49 | 0.29 | 0.45 | 0 | 1 |
| Income | 44.1 | 23.0 | 45.1 | 22.4 | 46.5 | 23.5 | 43.4 | 23.2 | 5 | 115 |
| Lowincome | 0.14 | 0.34 | 0.11 | 0.31 | 0.14 | 0.35 | 0.15 | 0.36 | 0 | 1 |
| University | 0.52 | 0.50 | 0.50 | 0.50 | 0.44 | 0.50 | 0.55 | 0.50 | 0 | 1 |
| Urban | 0.47 | 0.50 | 0.41 | 0.49 | 0.15 | 0.36 | 0.54 | 0.50 | 0 | 1 |
| ***TBE risk*** |  |  |  |  |  |  |  |  |  |  |
| TBE incidence in residence area | 1.11 | 4.05 | 0.84 | 0.87 | 12.80 | 9.95 | 0.01 | 0.02 | 0* | 41 |
| TBE risk residence area | 0.38 | 0.49 | 1.00 | 0.00 | 1.00 | 0.00 | 0.00 | 0.00 | 0 | 1 |
| High TBE risk residence area | 0.07 | 0.25 | 0.00 | 0.00 | 1.00 | 0.00 | 0.00 | 0.00 | 0 | 1 |
| TBE risk summerhouse | 0.17 | 0.37 | 0.22 | 0.42 | 0.20 | 0.40 | 0.14 | 0.34 | 0 | 1 |
| ***Behavioral risk*** |  |  |  |  |  |  |  |  |  |  |
| Outdoor in TBErisk area | 0.37 | 0.48 | 0.46 | 0.50 | 0.67 | 0.47 | 0.29 | 0.45 | 0 | 1 |
| Risk of tick bite at work | 0.10 | 0.29 | 0.11 | 0.32 | 0.17 | 0.38 | 0.08 | 0.27 | 0 | 1 |
| ***Experience with ticks*** |  |  |  |  |  |  |  |  |  |  |
| Tick bite ever | 0.68 | 0.47 | 0.74 | 0.44 | 0.85 | 0.36 | 0.63 | 0.48 | 0 | 1 |
| Tick disease experience | 0.45 | 0.50 | 0.51 | 0.50 | 0.55 | 0.50 | 0.41 | 0.49 | 0 | 1 |
| ***Knowledge and risk perceptions*** |  |  |  |  |  |  |  |  |  |  |
| Knowledge | 3.81 | 1.79 | 4.03 | 1.72 | 3.98 | 1.78 | 3.67 | 1.81 | 0 | 7 |
| Health risk tick bite | 0.42 | 0.49 | 0.45 | 0.50 | 0.62 | 0.49 | 0.38 | 0.48 | 0 | 1 |
| Low trust vaccine recommendations | 0.18 | 0.38 | 0.19 | 0.39 | 0.17 | 0.38 | 0.18 | 0.38 | 0 | 1 |

*In TBE-low risk areas, the minimum incidence is 0.11. In TBE-high risk areas, the minimum incidence is 5.1

**Age and Income coded as continuous variables**

Table C shows that age and income also are significantly associated with vaccination probability when coded as continuous variables

**Table C. Age and Income coded as continuous variables**

|  | (1) | (2) | (3) | (4) | (5) |
| --- | --- | --- | --- | --- | --- |
| VARIABLES | tbevaccinated | tbevaccinated | tbevaccinated | tbevaccinated | tbevaccinated |
|  |  |  |  |  |  |
| Female | 0.0158 | -0.00864 | -0.0162 | 0.0573 | -0.00334 |
|  | (0.0222) | (0.0213) | (0.0441) | (0.155) | (0.0211) |
| Age | -0.0115** | -0.0135*** | -0.0241*** | -0.0356 | -0.00632 |
|  | (0.00465) | (0.00442) | (0.00905) | (0.0365) | (0.00432) |
| Agesq | 0.000142*** | 0.000151*** | 0.000269*** | 0.000453 | 6.99e-05 |
|  | (4.58e-05) | (4.35e-05) | (8.94e-05) | (0.000354) | (4.27e-05) |
| Income | 0.00286*** | 0.00183*** | 0.00324*** | 0.0123*** | 0.000715 |
|  | (0.000474) | (0.000454) | (0.000951) | (0.00441) | (0.000443) |
| University | 0.0423* | 0.0103 | 0.0128 | -0.0991 | 0.00592 |
|  | (0.0224) | (0.0212) | (0.0429) | (0.163) | (0.0215) |
| Urban | 0.0612*** | 0.0660*** | -0.00361 | 0.225 | 0.0941*** |
|  | (0.0222) | (0.0220) | (0.0444) | (0.194) | (0.0220) |
| TBE incidence in residence area |  | 0.0124*** | 0.0286 | 0.00474 | -0.0656 |
|  |  | (0.00318) | (0.0238) | (0.00948) | (0.425) |
| TBE risk summerhouse |  | 0.108*** | 0.145** | 0.109 | 0.0913** |
|  |  | (0.0322) | (0.0577) | (0.197) | (0.0382) |
| Outdoor in TBE risk area |  | 0.195*** | 0.203*** | 0.583*** | 0.125*** |
|  |  | (0.0245) | (0.0425) | (0.114) | (0.0289) |
| Risk of tick bite at work |  | 0.115*** | 0.153* | 0.250 | 0.0776 |
|  |  | (0.0431) | (0.0811) | (0.155) | (0.0480) |
| Knowledge |  | 0.0462*** | 0.0631*** | 0.0565 | 0.0349*** |
|  |  | (0.00661) | (0.0138) | (0.0484) | (0.00663) |
| Tick bite ever |  | 0.0676*** | 0.0388 | 0.0978 | 0.0589** |
|  |  | (0.0238) | (0.0528) | (0.232) | (0.0234) |
| Tick disease experience |  | 0.0295 | 0.0743* | -0.0189 | 0.00869 |
|  |  | (0.0222) | (0.0432) | (0.178) | (0.0225) |
| Health risk tick bite |  | 0.0643*** | 0.0227 | 0.0804 | 0.0791*** |
|  |  | (0.0224) | (0.0438) | (0.171) | (0.0246) |
| Low trust in vaccine recommendations |  | -0.0531** | -0.111** | 0.131 | -0.0315 |
|  |  | (0.0243) | (0.0465) | (0.170) | (0.0243) |
|  |  |  |  |  |  |
| Observations | 1.526 | 1.526 | 485 | 100 | 941 |

Standard errors in parentheses; *** p<0.01. ** p<0.05. * p<0.1

## Control for omitted variable bias

We control for potential omitted variable bias in our estimation of TBE vaccine adoption (Table D). Columns 1-4 are identical with Table A above (but with a slightly smaller sample size). In Columns 5-8, we include four additional dichotomous variables: Swedishborn (=1 if born in Sweden); Health (=1 if respondent rates his/her health status as good or very good on a 1-5 scale); Hightrusthealthcare (=1 if respondent has rather or very high trust in Swedish health care); and Outdoorpet (=1 if respondent has a cat, dog or other pet which is not only inside the house).

When including these variables, the levels of significance for the variables included in Table A do not change.

**Table D. Control for omitted variable bias**

|  | (1) | (2) | (3) | (4) | (5) | (6) | (7) | (8) |
| --- | --- | --- | --- | --- | --- | --- | --- | --- |
|  | All respondents | Respondents living in TBE-risk areas | Respondents living in TBE-high risk areas | Respondents NOT living in TBE-risk areas | All respondents | Respondents living in TBE-risk areas | Respondents living in TBE-high risk areas | Respondents NOT living in TBE-risk areas |
|  | tbevaccinated | tbevaccinated | tbevaccinated | tbevaccinated | tbevaccinated | tbevaccinated | tbevaccinated | tbevaccinated |
| VARIABLES | mfx dydx | mfx dydx | mfx dydx | mfx dydx | mfx dydx | mfx dydx | mfx dydx | mfx dydx |
| Female | -0.0137 | -0.0366 | 0.0758 | -0.00510 | -0.0126 | -0.0428 | 0.0721 | -0.00181 |
|  | (0.0216) | (0.0440) | (0.150) | (0.0216) | (0.0217) | (0.0441) | (0.156) | (0.0214) |
| Age1830 | 0.0267 | 0.154* | 0.279 | -0.0211 | 0.0234 | 0.147* | 0.218 | -0.0240 |
|  | (0.0353) | (0.0821) | (0.234) | (0.0287) | (0.0349) | (0.0816) | (0.263) | (0.0277) |
| Age>65 | 0.0678*** | 0.165*** | 0.184 | 0.0216 | 0.0678*** | 0.175*** | 0.113 | 0.0194 |
|  | (0.0261) | (0.0560) | (0.158) | (0.0256) | (0.0263) | (0.0568) | (0.177) | (0.0253) |
| Low income | -0.0696*** | -0.106* | -0.523*** | -0.0230 | -0.0638** | -0.0947 | -0.581*** | -0.0142 |
|  | (0.0261) | (0.0578) | (0.117) | (0.0273) | (0.0270) | (0.0599) | (0.107) | (0.0285) |
| University | 0.0268 | 0.0418 | 0.0511 | 0.0120 | 0.0244 | 0.0418 | 0.114 | 0.00166 |
|  | (0.0211) | (0.0425) | (0.146) | (0.0215) | (0.0212) | (0.0423) | (0.156) | (0.0217) |
| Urban | 0.0712*** | 0.0127 | 0.174 | 0.0966*** | 0.0700*** | 0.0233 | 0.119 | 0.0879*** |
|  | (0.0223) | (0.0445) | (0.211) | (0.0225) | (0.0228) | (0.0452) | (0.224) | (0.0227) |
| TBE-incidence residence area | 0.0130*** | 0.0327 | 0.00736 | -0.0613 | 0.0131*** | 0.0341 | 0.00715 | -0.0156 |
|  | (0.00324) | (0.0240) | (0.00945) | (0.435) | (0.00324) | (0.0238) | (0.0103) | (0.434) |
| TBE-risk summerhouse | 0.117*** | 0.139** | 0.00437 | 0.108*** | 0.114*** | 0.142** | -0.0582 | 0.0994** |
|  | (0.0328) | (0.0564) | (0.196) | (0.0405) | (0.0328) | (0.0568) | (0.203) | (0.0394) |
| Outdoor in TBE-risk area | 0.194*** | 0.197*** | 0.543*** | 0.126*** | 0.192*** | 0.192*** | 0.562*** | 0.123*** |
|  | (0.0246) | (0.0429) | (0.116) | (0.0292) | (0.0247) | (0.0429) | (0.124) | (0.0290) |
| Risk of tick bite at work | 0.0915** | 0.108 | 0.195 | 0.0734 | 0.0920** | 0.0967 | 0.100 | 0.0738 |
|  | (0.0414) | (0.0766) | (0.167) | (0.0477) | (0.0414) | (0.0762) | (0.196) | (0.0470) |
| Knowledge | 0.0473*** | 0.0631*** | 0.0500 | 0.0361*** | 0.0467*** | 0.0651*** | 0.0447 | 0.0342*** |
|  | (0.00667) | (0.0139) | (0.0470) | (0.00671) | (0.00668) | (0.0138) | (0.0502) | (0.00669) |
| Tick bite ever | 0.0675*** | 0.0302 | 0.261 | 0.0582** | 0.0676*** | 0.0303 | 0.337* | 0.0594** |
|  | (0.0241) | (0.0538) | (0.193) | (0.0239) | (0.0241) | (0.0542) | (0.191) | (0.0235) |
| Tick disease experience | 0.0296 | 0.0833* | -0.0233 | 0.00637 | 0.0288 | 0.0866** | -0.0351 | 0.00610 |
|  | (0.0225) | (0.0439) | (0.172) | (0.0230) | (0.0225) | (0.0438) | (0.180) | (0.0227) |
| Healt risk tick bite | 0.0663*** | 0.0311 | 0.00539 | 0.0798*** | 0.0704*** | 0.0316 | -0.00221 | 0.0867*** |
|  | (0.0226) | (0.0436) | (0.160) | (0.0250) | (0.0228) | (0.0435) | (0.173) | (0.0252) |
| Low trust in vaccine recommendations | -0.0601** | -0.124*** | 0.0913 | -0.0327 | -0.0554** | -0.128*** | 0.130 | -0.0175 |
|  | (0.0243) | (0.0455) | (0.180) | (0.0249) | (0.0253) | (0.0450) | (0.190) | (0.0276) |
| Swedishborn |  |  |  |  | 0.0190 | -0.0579 | 0.0721 | 0.0499 |
|  |  |  |  |  | (0.0438) | (0.108) | (0.245) | (0.0357) |
| Health |  |  |  |  | 0.0202 | 0.0282 | -0.149 | 0.0225 |
|  |  |  |  |  | (0.0136) | (0.0264) | (0.106) | (0.0143) |
| High trust in health care |  |  |  |  | 0.00932 | -0.0173 | 0.272* | 0.0194 |
|  |  |  |  |  | (0.0228) | (0.0464) | (0.155) | (0.0229) |
| Outdoor pet |  |  |  |  | -0.00423 | 0.0784* | 0.0261 | -0.0361 |
|  |  |  |  |  | (0.0234) | (0.0472) | (0.172) | (0.0227) |
|  |  |  |  |  |  |  |  |  |
| Observations | 1,504 | 476 | 98 | 930 | 1,504 | 476 | 98 | 930 |

Standard errors in parentheses; *** p<0.01, ** p<0.05, * p<0.1
