# Supplementary material for: The Willingness to Pay for Vaccination against Tick-Borne Encephalitis and Implications for Public Health Policy: Evidence from Sweden
Source: PLoS One. 2015 Dec 7;10(12):e0143875. doi: 10.1371/journal.pone.0143875 (PMC4671589; doi:10.1371/journal.pone.0143875)
Supplement: S4 Text — (DOCX) [file pone.0143875.s006.docx]

# **S4 Text Prediction of the effects of a TBE vaccine subsidy**

In this document, we describe how we calculate the effects on vaccination rates of a possible TBE vaccine subsidy. Next, we control for potential hypothetical bias by only including those who state they were rather certain or very certain in their response to the WTP question. Finally, we separate those with a positive WTP from those with zero WTP in our calculations of predicted vaccination rates.

## Estimation of the effect of a possible subsidy

The current vaccination rate is derived from our survey. In TBE risk areas, the vaccination rate is 33% (CI95 30-37%).

We use the margins command in STATA to predict the effects of a possible subsidy on the demand for TBE vaccination in TBE risk areas.

We use *population mean* instead of sample mean for age (=49) female (=0.5) and income (=40600). For the other variables, we use sample means (S1 Text Willingness To Pay (WTP) estimation). We first make predictions for respondents in TBE risk areas and then for all unvaccinated respondents.

. quietly logit yesbidtbe bidtbe female age income university urban tberisk summerhouserisk outTBEarea worktickrisk knowledge tickbiteever diseaseexperience healthrisktickbite lowtrustvaccine if tberiskhome==1

. margins, at (bidtbe=(0 5.25)age=49 female=0.5 income=40.6) atmeans post

Adjusted predictions Number of obs = 389

1. The predicted vaccination rate among the unvaccinated in TBE risk areas at full subsidy, price zero = 68% (CI95 59-77%)
2. The predicted vaccination rate among the unvaccinated in TBE risk areas at 50% subsidy, price 525 = 46% (CI95 41-52%)

Based on our estimated vaccination rate of 33% among all respondents, we find the following predicted total vaccination rates, with a full subsidy and a 50% subsidy respectively, in TBE risk areas:

Full subsidy: Out of the 584 respondents in TBE risk areas, 389 are unvaccinated. With a full subsidy, 68% (0.68*389=265 respondents) of the unvaccinated would get vaccinated. This corresponds to 45% (265/584) of the total number of respondents in TBE risk areas. The new vaccination rate with the full subsidy would be 33%+45%=78%.

50% subsidy: With a 50% subsidy, 46.5% (0.465*389=181 respondents) of the unvaccinated respondents would get vaccinated. This corresponds to 31% (181/584) of the total number of respondents in TBE risk areas. The new vaccination rate with the 50% subsidy would be 33%+31%=64%.

This information is displayed in Table 3 in the paper.

*Predictions for all unvaccinated respondents*

. quietly logit yesbidtbe bidtbe female age income university urban tberisk summerhouserisk outTBEarea worktickrisk knowledge tickbiteever diseaseexperience healthrisktickbite lowtrustvaccine

. margins, at (bidtbe=(0 5.25)age=49 female=0.5 income=40.6) atmeans post

Adjusted predictions Number of obs = 1151

1. The predicted vaccination rate among all unvaccinated respondents at full subsidy, price zero = 67% (CI95 62-72%)
2. The predicted vaccination rate among the unvaccinated in TBE risk areas at 50% subsidy, price 525 = 43% (CI95 40-46%)

Based on our estimated vaccination rate of 25% among all respondents, we find the following predicted total vaccination rates with a full subsidy and a 50% subsidy:

Full subsidy: Out of the 1526 respondents in our sample, 1151 are unvaccinated (75%). With a full subsidy, 67% (771 respondents) of the unvaccinated would get vaccinated. This corresponds to 51% (771/1526) of the total number of respondents in TBE risk areas. The new vaccination rate with the full subsidy would be 25+51=76%.

50% subsidy: With a 50% subsidy, 43% of the unvaccinated (495) respondents would get vaccinated. 495/1526=32%. The new vaccination rate would be 25%+32%=57%.

These predicted vaccination rates are not included in the paper.

## Control for uncertainty of answer

We control for potential hypothetical bias in our estimates of effects of a vaccine subsidy in TBE risk areas by including only those who state they were rather certain or very certain in their response to the WTP question. When excluding the 56 respondents (out of 389) who were rather or very uncertain about their responses to the WTP question, we find:

- the same effect of a full subsidy on vaccination rates – 68%
- a 2 percentage point lower predicted vaccination rate – 44% compared to 46% - with a 50% subsidy

We conclude that our ex post control does not raise a concern that our estimates are influenced by hypothetical bias.

## Respondents with Zero WTP for a TBE vaccine

13% of the unvaccinated respondents living in areas with TBE risk state that they would not get vaccinated even if the vaccine was free of charge.

We separate these respondents from those respondents with a positive willingness to pay in an estimate of the predicted vaccination rate with a subsidized vaccine, using the same model as above.

We find that the predicted vaccination rate with a full subsidy in TBE risk areas increases by 1 percentage point to 79% and that the predicted vaccination rate with a 50% subsidy increases by 1 percentage point to 65%.

We conclude that analyzing those with zero WTP separately from those with positive WTP, compared to analyzing them jointly, gives similar predicted vaccination rates.
